# Prolonged Hypercalcemia Following Resection of Dysgerminoma: A Case Report

**DOI:** 10.1155/2009/956935

**Published:** 2009-12-13

**Authors:** Abigail Wald, Sumana Narasimhan, Lucybeth Nieves-Arriba, Steven Waggoner

**Affiliations:** ^1^Department of Pediatric Endocrinology, Rainbow Babies & Children's Hospital, 11100 Euclid Avenue, Cleveland, OH 44106, USA; ^2^Department of Gynecological Oncology, University Hospitals, 11100 Euclid Avenue, Cleveland, OH 44106, USA

## Abstract

*Background*. Hypercalcemia is a rare but potentially dangerous complication of pediatric cancer. Of the dysgerminoma cases reported to date, associated hypercalcemia is corrected within 2–7 days of tumor resection. *Case*. A 13-year-old female with an ovarian dysgerminoma was found to be hypercalcemic on presentation. Following dysgerminoma resection, moderate hypercaclemia persisted for 7 days and calcium remained mildly elevated for an additional 7 days. PTHrP was undetectable. Immunolocalization studies indicated that 1*α*-hydroxylase was expressed in dysgerminoma tissue but 1,25(OH)_2_D_3_ was not elevated. *Conclusion*. Persistently elevated calcium levels following tumor resection suggests that this case involves a previously undescribed mechanism. Elucidation of this mechanism may offer new insights into tumor biology and opportunities for therapeutic correction of hypercalcemia in this patient population.

## 1. Introduction

Humoral hypercalcemia of malignancy (HHM) is a rare but potentially dangerous complication of pediatric cancer. The incidence of pediatric malignancy-associated hypercalcemia is between 0.4 and 1.3% [1]. Ovarian dysgerminomas, which comprise two thirds of malignant ovarian neoplasms in children, are one of the most common ovarian cancers associated with hypercalcemia. This paraneoplastic disorder is usually related to tumor cell synthesis of parathyroid hormone-related peptide (PTHrP) or 1,25-dihydroxyvitamin D (1,25(OH)_2_D_3_) and typically resolves within one week of tumor resection [2–4]. 

We report a case of hypercalcemia associated with a dysgerminoma in which the etiology of hypercalcemia was not determined, in spite of extensive evaluation. This case is remarkable due to the prolonged duration of hypercalcemia following complete surgical resection of the tumor. 

## 2. Case

A 13-year-old female presented with a two-week history of anorexia, fatigue, fever, emesis, abdominal pain, and polyuria. There was no family history of endocrine neoplasia or parathyroid disease. Physical exam was notable for a large, firm mass extending from the pelvis to the left costal margin. CT scan and ultrasound revealed a left ovarian mass measuring 13 × 6.6 × 14 cm without evidence of metastases. 

Initial blood work revealed an elevated total calcium 14.9 mg/dL (9–10.6), normal alkaline phosphatase and albumin, as well as mildly elevated pancreatic enzymes. With the exception of an elevated LDH, tumor markers associated with ovarian cancer were normal. 

To correct the hypercalcemia (and associated pancreatitis), aggressive fluids and furosemide were administered. After several hours, total calcium decreased to 13.4 mg/dL (ionized 1.58 mmol/L). A dysgerminoma was suspected and a left salpingo-oophorectomy was performed. As lymph nodes were clinically suspicious, left pelvic and aortic lymph nodes were also resected. Pathologic evaluation confirmed a left ovarian dysgerminoma with an intact capsule and negative nodes and washings. 

**Figure 1:**
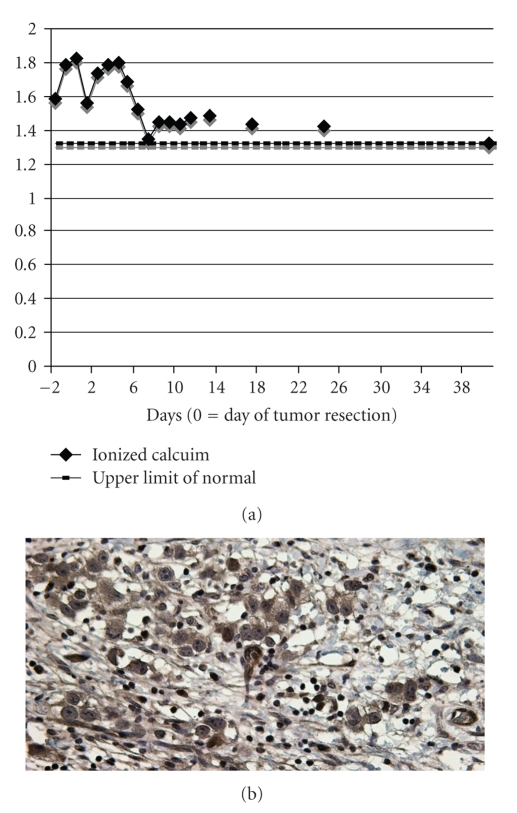
(a) Trend of ionized calcium after tumor resection. (b) Immunostaining for 1 *α*-hydroxylase was focally positive in tumor cell cytoplasm.

Postoperatively, IV fluids were continued with appropriate diuresis. Postoperative day (POD) 1, total calcium was 11.1 mg/dL (ionized 1.56 mmol/L). Unexpectedly, the patient's calcium began to rise (Figure 1(a)) and on POD 4, total calcium was 14.3 mg/dL (ionized 1.8 mmol/L). Calcium only stabilized (level <12 mg/dL) after furosemide was initiated. 

The persistence of hypercalcemia led to further investigation. Bone scan, SPECT, and PET imaging did not show evidence of metastases. Immunolocalization studies indicated that 1 *α*-hydroxylase was focally expressed in tumor cell cytoplasm (Figure 1(b)). Serum PTH and PTHrP were undetectable, Vitamin 1,25-dihyroxy was 19 pg/mL (15–75), vitamin D 25-hydroxy was 23 ng/mL (30–80), and urine phosphorus and calcium were low and high, respectively. On POD 6, serum calcium had decreased to near the upper range of normal. Fuorsemide and IV fluids were discontinued and the patient was discharged. Outpatient monitoring revealed stable calcium levels over time (Figure 1(a)).

## 3. Conclusion

Tumor associated hypercalcemia, not resulting from bone marrow invasion, is termed HHM, and is caused by factors secreted by tumors into the circulation. In most described cases, the etiologic factor identified is PTHrP, a compound similar to PTH, which causes increased bone resorption and renal calcium reabsorption. Although clinically PTHrP-induced hypercalcemia is similar to primary hyperparathyroidism, 1,25(OH)_2_D_3_ levels are low rather than high [2]. In the present case, low 1,25(OH)_2_D_3_ supports a PTHrP-related mechanism of hypercalcemia, however, undetectable PTHrP, low urine phosphorus and high urine calcium make this mechanism unlikely. 

1,25(OH)_2_D_3_ increases intestinal calcium absorption and, along with PTH, increases calcium resorption from bone and has been identified as another cause of HHM. The putative mechanism is increased expression of 1 *α*-hydroxylase in tumor cells and macrophages, an enzyme that catalyzes the synthesis of 1,25(OH)_2_D_3_ [2]. In this case, immunostaining of dysgerminoma tissue for 1 *α*-hydroxylase was positive. Although tumor expression of 1 *α*-hydroxylase is a plausible explanation, 1,25(OH)_2_D_3_ obtained on POD 1 was in the lower limits of normal making it unlikely that increased 1 *α*-hydroxylase resulted in persistent hypercalcemia. The half-life of 1,25(OH)_2_D_3_ is short, in the range 4–6 hours, but it is theoretically possible that intracellular effects persisted after serum levels of this vitamin D metabolite fell. 

Besides PTHrP and Vitamin D, production of prostaglandin or cytokines by tumor or immune cells may also play a role in HHM. These factors can increase bone resorption by inducing osteoclasts. We cannot exclude one of these factors or an unknown mediator contributing to the metabolic abnormality in this case. 

It is also conceivable, but unlikely, hypercalcemia was unrelated to the dysgerminoma. Coexisting conditions excluded were hypervitaminosis A and D, hyperparathyroidism, sarcoidosis, adrenal insufficiency, and hyperthyroidism. 

This case is unique as moderate hypercalcemia persisted 7 days following tumor resection and mild hypercalcemia persisted for an additional 7 days. In most reported cases of dysgerminoma associated hypercalcemia, calcium corrected within 2–7 days of tumor debulking [4]. 

Physicians should be aware of the association between hypercalcemia and pediatric cancer. Incomplete resolution of hypercalcemia for over a week following tumor resection suggests a previously undescribed mechanism. Elucidation of this mechanism may offer new insights into tumor biology and opportunities for novel therapeutics in this patient population.
